# 3D Printed Cell Culture Chamber for Testing the Effect of Pump-Based Chronic Drug Delivery on Inner Ear Tissue

**DOI:** 10.3390/biom12040589

**Published:** 2022-04-17

**Authors:** Jana Schwieger, Anna Sophie Frisch, Thomas S. Rau, Thomas Lenarz, Silke Hügl, Verena Scheper

**Affiliations:** 1 Department of Otorhinolaryngology, Head and Neck Surgery, Hannover Medical School, 30625 Hannover, Germany; frisch.anna@mh-hannover.de (A.S.F.); rau.thomas@mh-hannover.de (T.S.R.); lenarz.thomas@mh-hannover.de (T.L.); silke.huegl@tu-ilmenau.de (S.H.); scheper.verena@mh-hannover.de (V.S.); 2 Lower Saxony Center for Biomedical Engineering, Implant Research and Development (NIFE), Stadtfelddamm 34, 30625 Hannover, Germany; 3 Cluster of Excellence “Hearing4all” EXC 1077/2, 30625 Hannover, Germany

**Keywords:** auditory neurons, cochlear implant, cell culture chamber, neurite outgrowth, neurite guidance, ciliary neurotrophic factor, neurotrophin-3, pharmacotherapy, low cost, additive manufacturing

## Abstract

Cochlear hair cell damage and spiral ganglion neuron (SGN) degeneration are the main causes of sensory neural hearing loss. Cochlear implants (CIs) can replace the function of the hair cells and stimulate the SGNs electrically. The condition of the SGNs and their spatial distance to the CI are key factors for CI-functionality. For a better performance, a high number of neurons and a closer contact to the electrode are intended. Neurotrophic factors are able to enhance SGN survival and neurite outgrowth, and thereby might optimize the electrode-nerve interaction. This would require chronic factor treatment, which is not yet established for the inner ear. Investigations on chronic drug delivery to SGNs could benefit from an appropriate in vitro model. Thus, an inner ear inspired Neurite Outgrowth Chamber (NOC), which allows the incorporation of a mini-osmotic pump for long-term drug delivery, was designed and three-dimensionally printed. The NOC’s function was validated using spiral ganglion explants treated with ciliary neurotrophic factor, neurotrophin-3, or control fluid released via pumps over two weeks. The NOC proved to be suitable for explant cultivation and observation of pump-based drug delivery over the examined period, with neurotrophin-3 significantly increasing neurite outgrowth compared to the other groups.

## 1. Introduction

The standard therapy for patients with sensory hearing loss are cochlear implants (CIs). These electrically stimulating prostheses consist of two components: the external speech processor, needed for sound recording and for transforming the acoustic information into electrical impulses, and the stimulator-receiver, with the electrode carrier which is inserted into the scala tympani (ST), to stimulate the auditory nerve [1]. The cell bodies of the spiral ganglion neurons (SGNs) reside within the Rosenthal’s canal (RC) in the modiolus of the cochlea. Their peripheral processes (dendrites) project from their somata via the osseous spiral lamina towards the hair cells, which are located in the organ of Corti. The central processes (axons) of the SGNs form the auditory nerve and terminate in the cochlear nucleus of the brain. The performance of the CI depends, most importantly, on the amount of excitable SGNs and the proximity of the electrode to the SGNs [2]. Even though the developments in technical CI improvements are continuous, there are still limiting factors regarding the outcome of the CI. Among these are the degeneration of the SGNs, the loss of hair cells, and the retraction of peripheral nerve fibers of the SGNs. The preservation, and ideally regeneration, of the SGNs and their nerve fibers could promote further CI outcome improvements by an optimized nerve-electrode interface. A biological treatment, which supports the SGN survival and regeneration of the dendrites towards the electrodes, could potentially create the prerequisite for the implementation of an increased number of electrical channels for better speech perception and procession [1]. However, a local long-term treatment is challenging due to the isolated location of the SGNs. Drugs can be delivered to the inner ear through systemic, intratympanic, or intracochlear routes. Local application has several advantages over a systemic application such as bypassing the blood-labyrinth barrier (and reaching the target organ directly), higher drug concentration in the inner ear, and reduction of adverse systemic effects [3,4]. The intratympanic route is less effective than the intracochlear application because physiological barriers hinder it [5]. Besides a direct injection of a bolus into the cochlea [6,7], a chronic drug release could be achieved by mini-osmotic pumps [8], or in connection with the CI by an included delivery channel [9,10,11,12,13], a coating with factors [14,15,16,17], or an incorporation of drugs in the electrode carrier [18,19,20]. All these treatments have a risk of damaging the cochlear tissue and causing infections. Drug reservoirs need regular interventions for refills and the application of factor producing cells or gene transfection bear the risk of uncontrolled spread [21]. All these theories are the subject of current research for CI improvements. However, up to now, no satisfactory chronic inner ear pharmacotherapy for CI patients addressing the SGNs could be established. Several preclinical studies investigated SGN survival and found that, besides electrical stimulation, neurotrophic factors (NTFs) are able to increase neuronal survival [13,22]. For example, ciliary neurotrophic factors (CNTF) [23,24,25] and neurotrophin-3 (NT-3) have a positive effect on SGN survival and neurite outgrowth in vitro [26,27,28,29,30]. Additionally, NT-3 is able to attract SGN neurites in culture [27], whereas CNTF has not yet been tested for neurite guidance. However, none of the factors were observed for localized, long-term delivery in an inner ear culture system. Although a chronic factor treatment is considered to be a prerequisite for long-lasting regenerating effects on the SGNs and for induced guided neurite growth towards the electrode [31,32], there is currently no in vitro test system that allows the investigation of this issue. An adequate in vitro testing system for the inner ear is needed as a basis for the development of new treatment strategies, detection of suitable factors and a verification of their continuous biological activity when delivered chronically. Most commercially available microfluidic chambers, allowing for cell cultivation and the observation of neurite regeneration, focus on the investigation of dissociated cells. This certainly simplifies the observation of single neurons, but at the same time, destroys all physiological cell connections. In addition, all devices are only suitable for a rather short period of observation and none of them are geared to investigations on the cochlea. Therefore, we designed a Neurite Outgrowth Chamber (NOC) suitable to culture non-dissociated spiral ganglion explants (SGEs), allowing electrical stimulation via an included CI, and taking the anatomical structures of the inner ear into account. A first feasibility study proved the concept of this NOC for SGE culture in combination with a stimulating electrode [33]. To address the need for investigating long-term drug delivery to SGNs in vitro, this three-dimensionally (3D) printed NOC with a two-compartment design was adapted to an integrated drug delivery system. NT-3 and CNTF were chosen as known neuritogenic factors to validate the NOC for drug delivery to SGEs over two weeks via mini-osmotic pumps, which are suitable for in vivo application. Subsequently, neurite outgrowth was analyzed as proof of concept of this novel cell culture chamber to investigate drug effects on the intact spiral ganglion.

## 2. Materials and Methods

### 2.1. NOC Design and Manufacture

**Figure 1:**
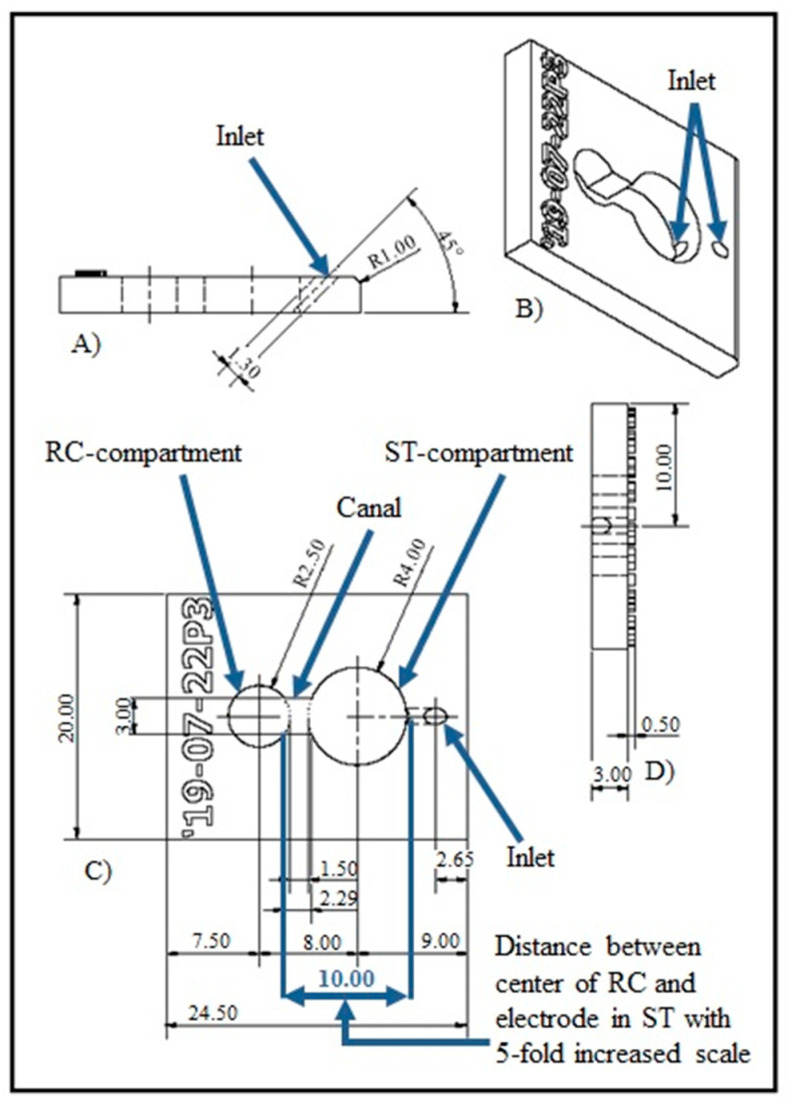
Overview image of the Computer Aided Design (CAD) data. The design of the Neurite Outgrowth Chamber (NOC) includes an inlet for drug delivery into the ST-compartment. Via the canal, those drugs can diffuse into the RC-compartment, housing the spiral ganglion explants (SGEs). (**A**) The position and angle (45°) of the inlet in the ST-compartment is illustrated in the side view of the NOC. (**B**) Both compartments and the inlet are presented in an oblique view. (**C**) The marked 10 mm distance represents a fivefold increased average of the reported distance of about 2 mm between the RC and a cochlear implant electrode in human cochleae (top view). (**D**) A rear view depicts the 3 mm height of the NOC. Dimensions in the drawing are given in mm.

**Figure 2:**
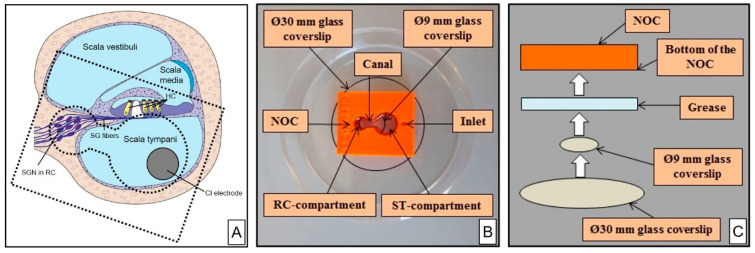
Illustration of a Neurite Outgrowth Chamber (NOC). (**A**) Drawn cross-section of the cochlea demonstrating the position of the ST-compartment and RC-compartment of the NOC (dashed lines, down scaled) in reference to the cochlea anatomy to illustrate the concept of the chamber. (**B**) Photography of the NOC (orange) being attached to the glass coverslips (circled with black lines) and placed within a Petri dish for subsequent cell culture. (**C**) Schematic drawing of the NOC inside view demonstrating the mount of the Ø9 mm glass coverslip below the canal and of the Ø30 mm glass coverslip to seal the whole NOC bottom mediated by the grease. HC = hair cells; RC = Rosenthal’s canal; SG = spiral ganglion; SGN = spiral ganglion neuron; ST = Scala tympani.

The NOCs were designed using Autodesk Inventor (Autodesk, San Rafael, CA, USA). The Computer Aided Design (CAD) data (Figure 1) included two compartments representing the Rosenthal’s canal (RC-compartment) and the scala tympani (ST-compartment) (Figure 2A) as previously described in a first feasibility study for the NOC including a CI electrode [33]. The RC-compartment (Ø5 mm) was used for placement of SGEs. By integrating a small inlet into the lateral wall of the ST-compartment (Ø8 mm), the NOC was modified for drug delivery (Figure 1C). The inlet (length of 2 mm) was designed with a slope of 45° for a simple and buckling-free insertion of the catheters for drug delivery (Figure 1A). The NOCs were manufactured with a height of 3 mm (Figure 1D) and an open top and bottom to enable tissue placement from the top and observation with an inverted microscope from the bottom during the experiments. A canal, allowing diffusion of the drug from the ST-compartment into the RC-compartment, connected both compartments in the chamber. The distance between the SGEs and the inlet was 10 mm (Figure 1C). This dimension was chosen based on the reported distance of 1.23 to 2.47 mm (~2 mm) between the center of the CI electrode array and the center of the RC in human inner ears [34]. For handling purposes, this dimension was increased by fivefold to allow manipulation of the SGEs in the NOC with forceps, sufficient medium supply, and to potentially support the formation of a diffusion gradient.

The NOCs were 3D printed (Ultimaker 2 and Ultimaker 2+; Ultimaker B.V., Utrecht, The Netherlands) using polylactic acid (PLA) (Innofil3D PLA; Innofil3D BV, Emmen, The Netherlands). Within one printing process, 11 NOCs were produced. Exemplary printing data of a printing session (11 NOCs) are presented in Table 1.

### 2.2. NOC Preparation for Cell Cultivation

To ensure the impermeability of the printed NOCs, they were covered with silicone (SYLGARD™ 184 Silicone Elastomer Base and Curing Agent; Fa. Dow Europe GmbH, Midland, MI, USA) by dip coating and drying overnight at room temperature. The bottom of the NOC was coated with grease (high vacuum grease; Dow Corning, Midland, MI, USA) to mount two circular glass coverslips on the bottom. A Ø9 mm glass coverslip for cell growth was placed below the canal followed by a Ø30 mm glass coverslip (both Menzel-Gläser, Braunschweig, Germany) to cover the whole NOC bottom (Figure 2B,C). The NOC was gently pushed against the glass coverslips to provide a liquid-tight seal mediated by the grease. The NOC, the glass coverslips, and the grease were decontaminated by UV radiation (Spectrolinker XL-1000 UV Crosslinker; Spectroline, Westbury, NY, USA) before use.

The inner glass bottom surface of the NOC (i.e., both compartments and the connecting canal) was coated with poly-DL-ornithine (0.1 mg/mL; Sigma Aldrich, Taufkirchen, Germany) and Natural Mouse Laminin (0.01 mg/mL; Invitrogen, Karlsruhe, Germany) to support cell adhesion and neuronal growth.

### 2.3. Setup for Pump-Based Drug Delivery

**Figure 3:**
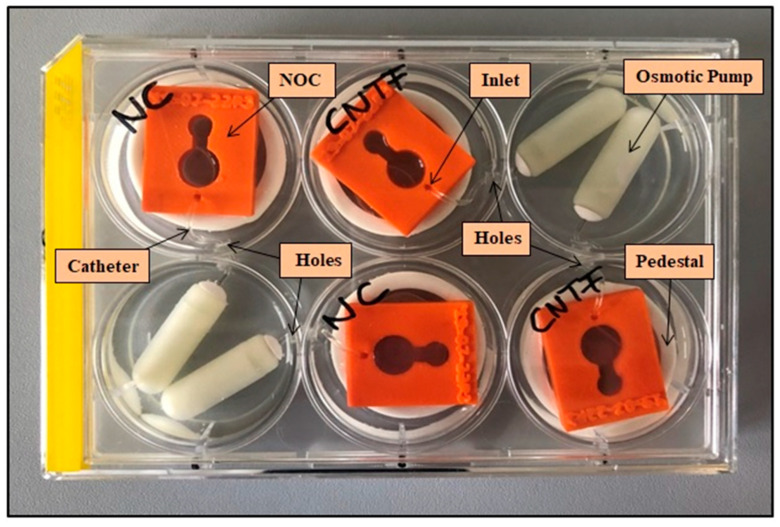
Experimental setup. Simultaneous cultivation of four Neurite Outgrowth Chambers (NOCs) (orange) placed on pedestals (white ring) in a modified 6 well plate. The NOCs are connected to the osmotic pumps by catheters that were threaded through the drilled holes in the walls of the wells.

Mini-osmotic pumps developed for in vivo experiments (Alzet model 2002; Durect Corporation, Cupertino, CA, USA; infusion rate 0.5 µL/h, 200 µL nominal reservoir, two weeks factor release) were used as drug source for long-term delivery. A 6 well tissue culture plate (TPP, Trasadingen, Switzerland) was modified to enable pump immersing next to the NOC for a parallel performance of N = 4 experiments (Figure 3). Small holes (Ø1.8 mm) were drilled into the multi-well plate at a height of 1 cm to allow a connection between the separated NOCs and osmotic pumps without compression of the catheter. The catheters (Rat Femoral Catheter; Durect Corporation, Cupertino, CA, USA; outer diameter of 1.02 mm) were cut into 3 cm long pieces, linked to the pumps, threaded through the drilled holes, and inserted in the inlet of the NOC. Two of the 6 wells served as a depot for two mini-osmotic pumps each, whereas the other four wells hosted the NOCs. Wells were filled with saline (NaCl 0.9%; B. Braun, Melsungen, Germany) until the pumps were completely covered to ensure a continuous drug release. Due to the drilled holes, saline diffused to all wells of the plate and prevented an evaporation of the medium in the NOCs. A placement on 3D printed pedestals (white PLA, height: 11 mm; thickness: 7 mm; diameter: 30 mm) elevated the NOCs above the saline level.

### 2.4. Validation

To prove the feasibility of the NOC for investigation of pump-based, long-term drug delivery for neurite outgrowth of SGEs, CNTF (recombinant human CNTF; Novoprotein, Summit, USA) and NT-3 (recombinant human NT-3; Peprotech, Cranbury, NJ, USA) were chosen as known neuritogenic factors, with NT-3 also having a documented neurite attraction potential. Both factors were compared to negative controls (NCs) receiving 90% artificial human perilymph (AP, see below) and 10% distilled water via catheters. Pumps and catheters of the test groups were filled with 90% AP and 10% NTF solution (NTF stock solution in distilled water: 10 µg CNTF/mL, 7.5 µg NT-3/mL; further diluted in AP for pump filling: 1.0 µg CNTF/mL, 750 ng NT-3/mL). AP consisted of 145 mM sodium chloride (Merck, Darmstadt, Germany), 2.7 mM potassium chloride (Merck, Darmstadt, Germany), 2.0 mM magnesium sulphate heptahydrate (Merck, Darmstadt, Germany), 1.2 mM calcium chloride dihydrate (Sigma Aldrich, Taufkirchen, Germany), 5.0 mM HEPES (Invitrogen, Karlsruhe, Germany), and human serum albumin (1 µg/µL; PAN BIOTECH, Passau, Germany) [13]. The NTF concentrations were chosen based on the following calculations: multiplying the CNTF concentration of 1 ng/µL with a pump infusion rate of 0.5 µL/h, 0.5 ng CNTF were released into the 150 µL culture medium in one hour. Without consideration of the cells’ metabolism, a CNTF concentration of 100 ng/mL (=15 ng/150 µL) was reached after 30 h. This concentration is known to support neurite outgrowth [23]. The basis for the NT-3 concentration is the publication of Wittig et al. [27] detecting a neurite attraction induced by 25 ng/mL NT-3 at a minimal delivery rate of 15 µL/h. Due to the lower delivery rate of 0.5 µL/h of the used mini-osmotic pumps, they were filled with 750 ng/mL NT-3 to achieve the same amount of NTF delivery.

To observe the effect of the cultivation in the NOC connected to a pumping system, the NOC-cultured cells were also compared with a second control group that was not connected to pumps (no pump (NP)). The NPs received a plain culture medium (see below). To test for negative effects of concentrated NTF (NT-3 only) due to the long delivery period during the experiments and to promote a diffusion gradient between pump and cells, additional testing groups with daily medium change (MC) were included (NT-3 MC, NC MC, NP MC). All treatment conditions and individual, evaluable repetitions are listed in Table 2.

### 2.5. Preparation of Spiral Ganglion Cells

All experiments were performed in accordance with the German Law on Protecting Animals (§4) and to the European Directive 2010/63/EU for protection of animals used for experimental purposes. The tissue harvesting was registered (No. 2016/118) with the local authorities (Laboratory Animal Science). The detailed preparation procedures for SGE harvesting have already been described [23]. In short, early postnatal Sprague Dawley rats (P2-4) were decapitated, the skulls were opened, the temporal bones were separated, and transferred into a Petri dish containing ice-cold Ca^2+^/Mg^2+^-free D-phosphate-buffered saline (PBS) (Gibco Invitrogen, Karlsruhe, Germany). The cochlea was exposed by removing the bony cochlear capsule under a microscope (Leica KL 1500 LCD; Leica, Wetzlar, Germany). After transferring the membranous labyrinth, the entire spiral ganglion (SG) was detached from the modiolus and separated from the organ of Corti and the stria vascularis. The SGs were stored in PBS on ice until they were cut into five pieces (spiral ganglion explants, SGEs) of approximate equal length and transferred into the NOCs.

### 2.6. Cultivation of SGEs

**Figure 4:**
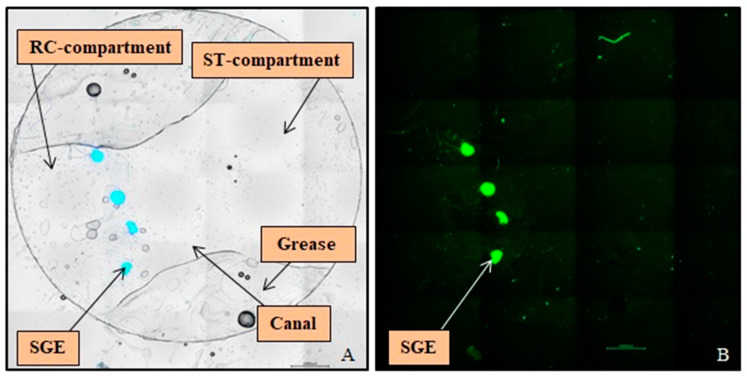
Overview pictures of Spiral Ganglion Explants (SGEs) on the Ø9 mm Glass Coverslip after Immunocytochemistry. (**A**) In the merged bright field image four SGEs stained against DAPI (blue) are visible, cultured on the bottom of the Rosenthal’s canal-compartment in front of the canal. The used grease for sealing the Neurite Outgrowth Chamber left an imprint on the glass coverslip. (**B**) The fluorescence image illustrates the same SGEs labeled for neurofilament (green), which was used for analysis of neurite outgrowth. Scale bars: 1000 µm.

Each NOC was filled with 150 µL culture medium consisting of Panserin 401 (PAN BIOTECH, Passau, Germany) supplemented with insulin (8.7 µg/mL; Biochrom, Berlin, Germany), penicillin (30 U/mL; Biochrom, Berlin, Germany), glucose (0.15%; B. Braun, Melsungen, Germany), PBS (0.172 mg/mL), HEPES-buffer solution (23.43 µM; Invitrogen, Karlsruhe, Germany), N2-supplement (0.1 µL/mL; Invitrogen, Karlsruhe, Germany), as well as 10% Fetal Calf Serum (Invitrogen, Karlsruhe, Germany). The SGEs were placed on the bottom of the RC-compartment in front of the canal (Figure 4A) with their peripheral side facing the pump and arranged in a base to apex order. After positioning the SGEs, the NOCs were stored at room temperature for 30 min without moving to allow proper tissue adherence before they were transferred to the incubator. The NOCs were pre-cultivated in a serum condition to support the beginning of cell growth after tissue preparation and for a tight connection of the SGEs on the surface of the coated glass coverslips for one day in the incubator (CB 150 E3; Binder, Tübingen, Germany; 37 °C, 5% CO_2_, 95% humidity). On the same day, the culture plate, osmotic pumps, and catheters were prepared to ensure pre-incubation of the pumps at 37 °C for one day according to the manufacturer’s instructions. The next day, the culture medium was changed in all NOCs to medium without serum to test a serum-unaffected impact of the NTFs versus factor free medium. The NOCs of the NC, CNTF, NT-3, NC MC, and NT-3 MC groups were transferred into the culture plate and connected to the pumps. The NOCs of the NP and NP MC groups were kept in Petri dishes and cultured without pumps. All experimental groups were cultivated under the same conditions in the incubator for an additional four days. The ability of the osmotic pumps to release their substances for 14 days enabled the performance of at least two independent experiments in a row. The first set of NOCs was removed from the osmotic pumps after four days and replaced with freshly prepared and one day pre-cultivated ones for the next experiment in the second week of drug release. After five days of cultivation in total, the SGEs were fixed with 4% paraformaldehyde (Merck, Darmstadt, Germany) for 10 min at room temperature and carefully washed three times with PBS to avoid detachment of the SGEs. Both glass coverslips were removed from the NOCs and the small coverslip with the attached SGEs was transferred into a 48 well plate (Thermo Fisher Scientific, Roskilde, Denmark) for subsequent staining procedure.

### 2.7. Immunocytochemistry

To analyze number, length, and orientation of the neurites, the neurons of the SGEs were specifically stained. Cells were permeabilized using 0.5% Triton X-100 (Sigma Aldrich, Taufkirchen, Germany) in PBS for 15 min, washed three times with PBS, and blocked for one hour with blocking solution consisting of PBS, 10% Fetal Calf Serum, 5% Bovine Serum Albumin (Sigma Aldrich, Taufkirchen, Germany), and 0.1% Triton X-100. As primary antibody, the Anti-Neurofilament heavy polypeptide antibody (ab4680; Abcam, Cambridge, United Kingdom; host species: chicken, diluted 1:10,000–1:5000 in blocking solution) was added to the cells for one hour at room temperature. Subsequently, the cells were rinsed with PBS thrice for 10 min. The secondary antibody Goat Anti-Chicken IgY H&L (DyLight 488, 1:200; ab96947; Abcam, Cambridge, UK) and DAPI (1:10; AppliChem GmbH, Darmstadt, Germany), for nucleus labeling, diluted in blocking solution, were added for one hour in darkness at room temperature. Finally, remaining unbound secondary antibodies were washed off with PBS for 10 min thrice and labeled cells were stored in PBS.

### 2.8. SGE Analysis

**Figure 5:**
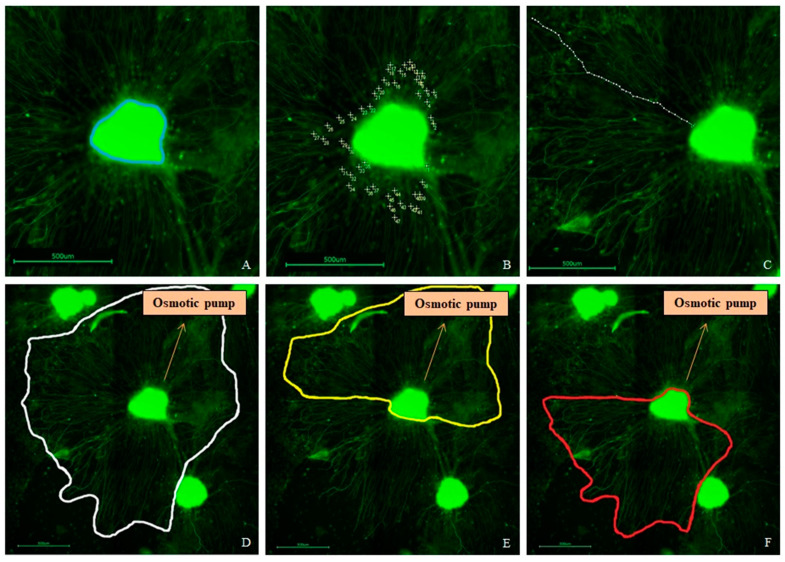
Examples of an analysis of the Spiral Ganglion Explant (SGE) parameters after neurofilament labeling (green). (**A**) Measured SGE area (blue line). (**B**) Counted number of outgrown neurites. (**C**) Measured length of one of the analyzed 40 longest outgrown neurites. (**D**) Neurite outgrowth area determined by connecting the ends of all neurites (white line). (**E**) Area of neurites grown towards the osmotic pump (yellow line). (**F**) Area of neurites grown opposed to the pump (red line). Included arrows indicate the direction towards the osmotic pump. Scale bars: 500 µm.

For analysis of the SGEs and their neurites, pictures were taken with a fluorescence microscope (Keyence BioRevo BZ-9000E; Keyence, Neu-Isenburg, Germany) in combination with the BZ II Viewer and Analyzer software. An overview picture series was taken with a 4× objective and composed for orientation to count the number of SGEs being attached after five days of cultivation (Figure 4A). For analysis of neurite outgrowth, picture series of each SGE with the associated regenerated neurites were taken with the 10× objective and composed. ImageJ 1.52 n (National Institutes of Health, Bethesda, MD, USA) was used for measurements and counting. The area of each SGE (Figure 5A), the number of all outgrown neurites (Figure 5B), and the length of the 40 longest regenerated neurites per SGE, if existent, (Figure 5C) were analyzed. Since the NOC was designed with a localized drug release in the ST-compartment, the neurites could be analyzed for their direction, which may indicate a neurite attraction by the factors. Therefore, the number of neurites was additionally divided into two directions to display the orientation of the neurite outgrowth: they either grew towards the mini-osmotic pump or opposed to it. The orientation was set depending on the end of each neurite. The area covered by regenerated neurites was determined by connecting the ends of all neurites around each SGE in total and subtracting the respective SGE area (Figure 5D). This area was also divided into the two mentioned directions for neurite orientation and the respective SGE area was subtracted as well (Figure 5E,F).

### 2.9. Statistical Analysis

The statistical analysis was performed using GraphPad Prism 5.02 (GraphPad Software, Inc., San Diego, CA, USA). Data were checked for normal distribution using the D’Agostino and Pearson normality test. No normal distribution was observed and the mean ± the standard deviation (SD) of the measured data were compared. No multiple comparison test was applied but selected experimental groups that correspond to each other were analyzed using the Mann Whitney U-test. Statistical significance was considered at *p* values less than 0.05 and is depicted in the graphs as follows: ns = no significant difference; *p* < 0.05 = *; *p* < 0.01 = **; *p* < 0.001 = ***.

## 3. Results

### 3.1. NOC Manufacture

Three-dimensionally printing the NOCs with PLA using the fused deposition modeling technology enables quick and cheap production. Each printing process produces 11 NOCs within 5 h 25 min. Due to the PLA’s stability and the possibility of repeated decontamination after each experiment, the chambers are reusable. The quick and simple coating with silicone prevents the medium from seeping into the NOC walls. The coating can be peeled off and renewed for subsequent experiments.

### 3.2. Cultivation of SGEs in the NOCs

**Figure 6:**
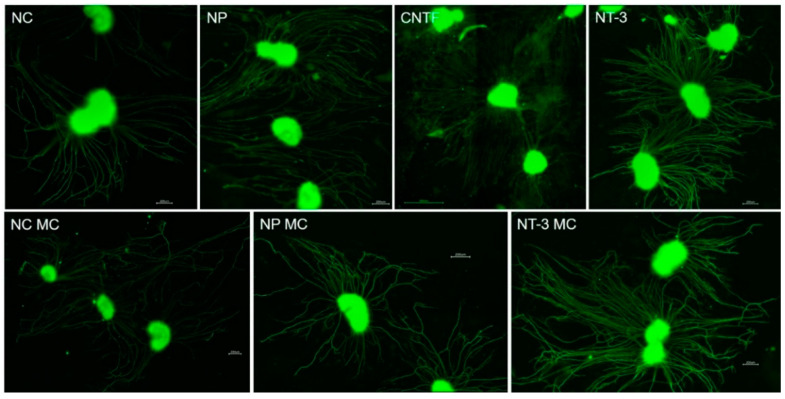
Fluorescence images of Spiral Ganglion Explants (SGEs) observed in different conditions exemplarily showing the SGE area and neurite outgrowth. Scale bars: 200 µm (CNTF: 500 µm).

The setup for drug delivery with mini-osmotic pumps over two weeks in the modified six well plate for four simultaneous pump-NOC combinations proves to be very suitable for the intended investigation of pharmacotherapeutic effects on SGEs. Changing the NOCs for the second week of drug release with new SGEs is performed without any problems. The compact construction and the silicone coating of the NOCs ensure the impermeability for the medium, and the grease mediates a tight connection between NOCs and glass coverslips in the experiments. Extensive outgrowth of non-neuronal cells in a radius around the SGEs and neurite extension of the SGN, regardless of treatment, are generally observed (Figure 6). An adherence of the SGEs on the glass coverslip is supported by cutting the SGs into five parts, by coating the glass with ornithine and Laminin, by leaving the NOCs at room temperature for 30 min without any movements after SGE positioning, and by pre-cultivating the SGEs in serum condition for one day. Nevertheless, not all SGEs adhere to the surface in front of the canal. Some SGEs attach in the canal or ST-compartment and some detach during fixation or staining due to incomplete adherence. This is why two to five SGEs per NOC are present after cultivation.

### 3.3. SGE Analysis

**Figure 7:**
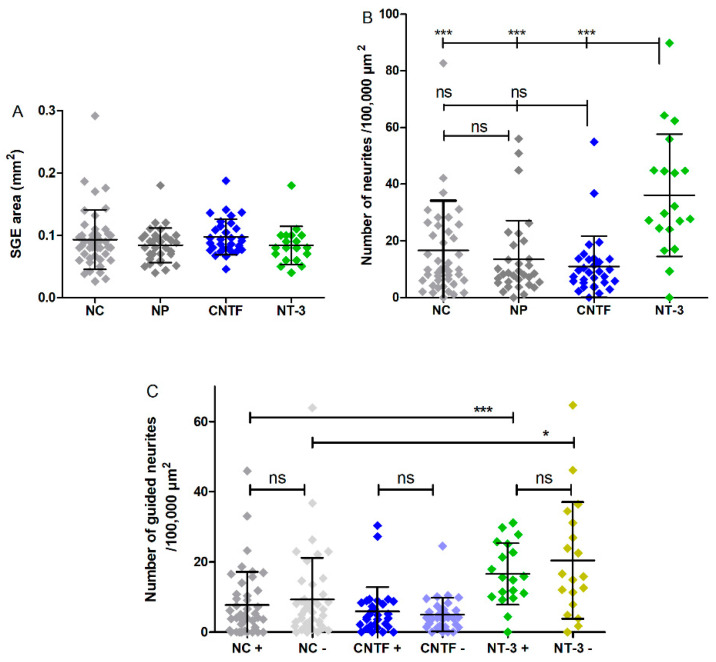
Explant area, number of neurites, and number of guided neurites per condition (without medium change). (**A**) The area of the cultured explants does not vary significantly between the experimental groups. (**B**) The mean number of neurites per 100,000 µm^2^ explant area independent of the direction of outgrowth is significantly increased in the NT-3 group compared to the NC, to the NP, and to CNTF. (**C**) The mean numbers of grown neurites dependent on the direction (+ or −) per 100,000 µm^2^ explant area of the NC, CNTF, and NT-3 groups are plotted. No guided neurite outgrowth is detectable. The mean number of neurites in the NT-3 + group is significantly higher than in the NC +. Additionally, a significant increase is visible between NT-3 − and NC −.

The number of adhered SGEs is counted and the area of the SGEs is measured. The explant areas range from a minimum of 0.02 mm^2^ to a maximum of 0.29 mm^2^ in all groups (Figure 7A). There is no significant difference in the explant areas between the groups with means of 0.09 ± 0.04 mm^2^ (NC), 0.08 ± 0.02 mm^2^ (NP), 0.09 ± 0.02 mm^2^ (CNTF), and 0.08 ± 0.03 mm^2^ (NT-3).

### 3.4. Neurite Outgrowth Analysis

Neurite outgrowth is assessed in terms of number, length, and coverage area. The NOC setup with the drugs being released by the pump at the lateral wall of the ST-compartment shall enable the formation of a diffusion gradient of the factors by the distance to be overcome to the SGEs in the RC-compartment. It also allows the analysis of the orientation of grown neurites in direction to the factor source. This provides the prerequisites for examining a neurite guidance.

#### 3.4.1. Number of Neurites

The number of neurites per 100,000 µm^2^ reach maximum values of 82.67 (NC), 56.00 (NP), 54.89 (CNTF), and 89.77 (NT-3) after five days of cultivation. SGEs co-cultured with NT-3-delivering pumps have a significantly higher number of neurites per 100,000 µm^2^ with a mean of 36.08 ± 21.56 than the NC (mean: 16.59 ± 17.55), the NP (mean: 13.40 ± 13.83), and CNTF (mean: 10.96 ± 10.76) with *p* < 0.001 for all comparisons (Figure 7B).

#### 3.4.2. Guided Neurite Outgrowth

Neurite orientation in relation to the osmotic pump is detected by manually counting how many neurites grow towards the pump (+) or opposed to it (−). For direct comparison, the number of neurites is calculated per 100,000 µm^2^ explant area. As graphically illustrated in Figure 7C, the mean number of neurites in the NT-3 + group (16.62 ± 8.76) is significantly increased compared to the NC + group (7.77 ± 9.43, *p* < 0.001). In addition, significantly more neurites grow in the NT-3 − group (20.43 ± 16.61) than in the NC − (9.33 ± 11.88, *p* < 0.05). There is no significant difference between NT-3 + and NT-3 − as well as between CNTF + (5.93 ± 6.87) and CNTF − (5.03 ± 4.79). The NP is not attached to osmotic pumps and is therefore excluded from analysis of directed neurite growth.

#### 3.4.3. Neurite Length

For analysis of the neurite length, the 40 longest regenerated neurites per SGE are measured if existent and traceable. Since two to five SGEs per NOC are present after cultivation, 80 to 200 neurites are measured per NOC, resulting in N = 551 (NC), N = 347 (NP), N = 303 (CNTF), and N = 476 (NT-3) analyzed neurites. Table 3 depicts the measured neurite lengths (minimum, maximum, and mean) independent of the direction of outgrowth.

**Figure 8:**
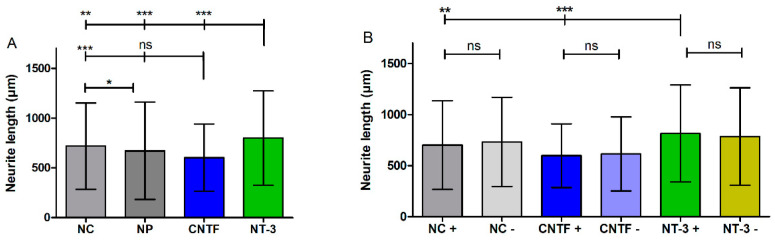
Neurite length with and without directional analysis (without Medium Change). (**A**) Mean and SD of neurite lengths of all four experimental groups independent of the direction of outgrowth. Here, the neurite lengths are significantly longer in the NT-3-treated group than in the NC, CNTF, and NP groups. NC has significantly longer neurites compared to CNTF and to NP. (**B**) The bar charts illustrate the mean lengths of grown neurites towards (+) or opposed to the osmotic pump (−). Neurites of the NT-3 + group grow significantly longer than neurites of the CNTF + group and neurites of the NC + group. No significant differences are observed within the groups.

In Figure 8A the means of neurite lengths, regardless of the direction, are displayed. NT-3 shows the longest neurites with a mean length of 799 ± 475 µm, which is a significant increase compared to the NC (mean: 717 ± 434 µm, *p* < 0.01), to the NP (mean: 670 ± 489 µm, *p* < 0.001), and to CNTF (mean: 603 ± 336 µm, *p* < 0.001). For further analysis of neurite orientation, the lengths of neurites that were either grown towards the pump (+) or away from it (−) are compared as well; therefore, the measured neurite lengths (Figure 8A) are assigned to both directions (Figure 8B, Table 4). Neurites of the NT-3 + group (mean: 814 ± 474 µm) grow significantly longer than neurites of the CNTF + group (mean: 597 ± 311 µm, *p* < 0.001) and neurites of the NC + group (mean: 701 ± 433 µm, *p* < 0.01). No significant differences are observed between NT-3 + and NT-3 − (mean: 784 ± 476 µm), between CNTF + and CNTF − (mean: 614 ± 363 µm), or between NC + and NC − (mean: 732 ± 436 µm).

#### 3.4.4. Neurite Coverage Area

**Figure 9:**
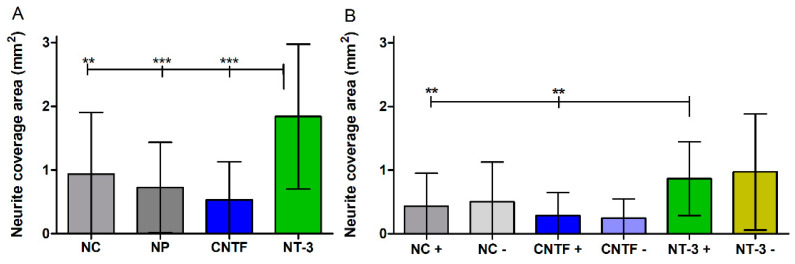
Neurite coverage area with and without directional analysis (without medium change). (**A**) The area covered with regenerated neurites is determined by marking the outline of the outgrown neurites and subtracting the respective spiral ganglion explant area. The outgrowth area independent of the direction is significantly increased in the NT-3 group compared to NC, NP, and CNTF. (**B**) Mean neurite outgrowth areas dependent on direction are plotted. A significant increase of the area in the NT-3 + group compared to NC + and to CNTF + is observed, whereas no differences are detectable within the groups.

By connecting the ends of all neurites around the SGE independent of the direction and subtracting the respective SGE area, the area covered by outgrown neurites is determined. As shown in Figure 9A, the means of outgrowth areas reach from 1.83 ± 1.13 mm^2^ (NT-3) to 0.93 ± 0.96 mm^2^ (NC) to 0.70 ± 0.71 mm^2^ (NP) to 0.53 ± 0.59 mm^2^ (CNTF). The neurite coverage area of the NT-3 group is significantly larger than the areas of the NC group (*p* < 0.01), the NP group (*p* < 0.001), and the CNTF group (*p* < 0.001). The area covered by outgrown neurites dependent on the direction is determined by connecting the ends of neurites around the SGE that either grow towards (+) or opposed (−) to the pump and subtracting the respective SGE area. In Figure 9B, the means and SD of neurite coverage areas dependent on the direction of neurite outgrowth are displayed and vary in the groups from 0.97 ± 0.91 mm^2^ (NT-3 −) to 0.86 ± 0.58 mm^2^ (NT-3 +), from 0.50 ± 0.62 mm^2^ (NC −) to 0.43 ± 0.52 mm^2^ (NC +), and from 0.28 ± 0.36 mm^2^ (CNTF +) to 0.24 ± 0.30 mm^2^ (CNTF −). The neurite outgrowth area of NT-3 + is significantly increased compared to the NC + and CNTF + with *p* < 0.01.

### 3.5. Influence of Medium Change on NOC Results

**Figure 10:**
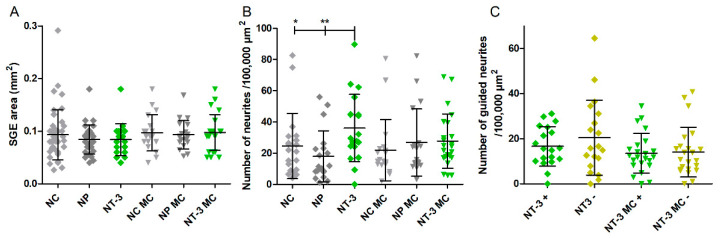
Explant area, number of neurites, and number of guided neurites per condition with medium change (MC). (**A**) No differences in spiral ganglion explant (SGE) area of the groups with and without MC are detected. (**B**) The number of neurites per 100,000 µm^2^ SGE area independent of the orientation does not differ within the MC groups. In contrast, significantly more neurites grow in the NT-3 group without MC compared to the NC and the NP. (**C**) In this chart, the mean numbers of neurites grown towards (+) or opposed to the osmotic pump (−) per 100,000 µm^2^ SGE area of the NT-3 groups with and without MC are compared. No significant changes are observed.

Chronic pump-based delivery may result in an accumulation possibly leading to an overdose and a steady state of drug concentration, which prevents the maintenance of the concentration gradient required for guided neurite outgrowth. To avoid a prolonged drug accumulation, medium change (MC) was performed daily in an additional series of experiments. The SGE areas are equal in all compared conditions (Figure 10A) with mean values of 0.09 ± 0.04 mm^2^ (NC), 0.08 ± 0.02 mm^2^ (NP), 0.08 ± 0.03 mm^2^ (NT-3), 0.09 ± 0.03 mm^2^ (NC MC), 0.09 ± 0.02 mm^2^ (NP MC), and 0.09 ± 0.03 mm^2^ (NT-3 MC). The number of neurites per 100,000 µm^2^ explant area, independent of the direction, does not differ between the groups with MC (Figure 10B). This is in contrast to the detected significant differences for the corresponding groups without MC, since NT-3 shows significantly more neurites than NC (*p* < 0.05) and NP (*p* < 0.01). The mean numbers of 24.59 ± 20.93 (NC), 17.93 ± 16.34 (NP), 36.08 ± 21.56 (NT-3), 21.90 ± 19.57 (NC MC), 26.75 ± 21.56 (NP MC), and 27.73 ± 17.41 (NT-3 MC) are presented in Figure 10B. After five days of cultivation, maximum numbers of 82.67 (NC), 56.00 (NP), 89.77 (NT-3), 80.62 (NC MC), 82.24 (NP MC), and 68.94 (NT-3 MC) outgrown neurites are reached. The number of guided neurites does not differ between the NT-3 groups with and without MC (Figure 10C) as the mean numbers reach from 16.62 ± 8.76 (NT-3 +) to 20.43 ± 16.61 (NT-3 −) and from 13.51 ± 8.79 (NT-3 MC +) to 14.05 ± 11.01 (NT-3 MC −).

**Figure 11:**
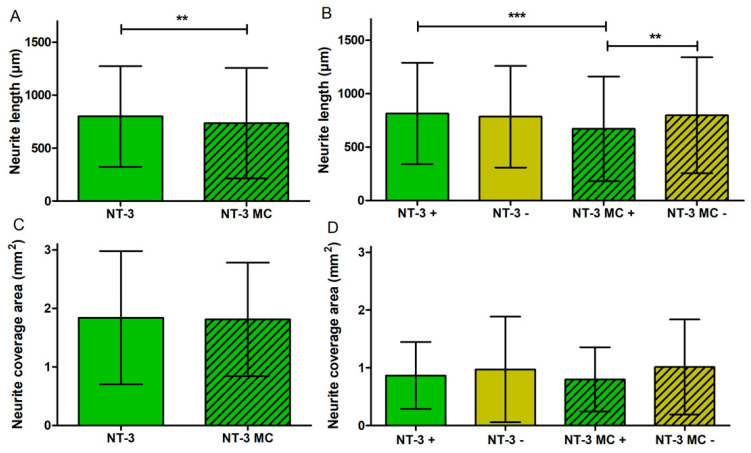
Neurite length and coverage area with and without directional analysis with medium change (MC). (**A**) Mean neurite lengths of NT-3 and NT-3 MC independent of the direction are illustrated. NT-3 shows a significantly higher length compared to NT-3 MC. (**B**) Mean neurite lengths of NT-3 and NT-3 MC dependent on the direction of outgrowth are depicted. Compared to NT-3 MC +, the neurites of NT-3 + and NT-3 MC − are significantly longer. (**C**) The neurite coverage area independent of the direction of outgrowth does not differ between NT-3 and NT-3 MC. (**D**) The mean neurite outgrowth areas dependent on the direction show no significant differences between the NT-3 groups with or without medium change.

Further examination of the neurite length is presented in Figure 11A, B where the NT-3 groups with and without medium change are compared. The neurite lengths independent of the direction are significantly increased in the NT-3 group (N = 476; mean: 799 ± 475 µm) compared to the NT-3 group with MC (N = 513; mean: 734 ± 520 µm, *p* < 0.01) (Figure 11A). Depending on the direction, significant differences in neurite lengths are detected and shown in Figure 11B. In the NT-3 + group (mean of 814 ± 474 µm), significantly longer neurites grow out compared to the NT-3 MC + group (mean of 671 ± 490 µm; minimum: 38 µm; maximum: 3770 µm, *p* < 0.001). In addition, the neurites of the NT-3 MC − group (mean of 798 ± 542 µm; minimum: 31 µm; maximum: 2825 µm) are significantly longer than the neurites of the NT-3 MC + group (*p* < 0.01). To examine the effect of medium change, the neurite outgrowth area independent of the direction is compared between the NT-3 group and the NT-3 MC group (Figure 11C). The mean values (NT-3: 1.83 ± 1.13 mm^2^; NT-3 MC: 1.81 ± 0.96 mm^2^) do not differ from each other. Additionally, the neurite coverage areas dependent on the direction of outgrowth of NT-3 and NT-3 MC are compared in Figure 11D. The statistics show no significant differences as the means vary from 0.86 ± 0.58 mm^2^ (NT-3 +) to 0.97 ± 0.91 mm^2^ (NT-3 −) and from 0.79 ± 0.55 mm^2^ (NT-3 MC +) to 1.01 ± 0.82 mm^2^ (NT-3 MC −).

## 4. Discussion

The NOC was previously described in a first proof-of-concept trial for electrical stimulation of SGEs with a CI electrode [33]. In the present study, its adaption for drug delivery and a simple setup with mini-osmotic pumps for long-term factor release are described. Its capability is validated with two test factors known to support neurite outgrowth. A sufficient number of excitable SGNs is a prerequisite for the function of a CI and a reduced distance between neurons and electrodes could further improve its function. Thus, research on supporting the SGN survival and bridging the distance by induced neurite growth of SGNs is necessary. Both goals could be achieved by treatment with NTFs. Due to the (life)long use of the CI by patients and the long spatial distance to be bridged by regenerating neurites, chronic treatment instead of bolus application with supporting factors is aimed for this optimization. In this context, a continuing biological effectiveness of administered factors has to be ensured before being used in vivo. SGN culture is a common method in the research and development of pharmacotherapies for the inner ear [35]. Up to now, there is no in vitro test system established that would enable the investigation of chronic factor treatment to SGEs or would be combinable with a common in vivo drug release system, matrices for neurite growth support, or electrodes for electrical stimulation. The currently used neuronal culture devices are either designed to cultivate dissociated neurons and have no open top allowing SGE placement (Campenot Chamber [36], microfluidic multi-compartment device by Taylor et al. [37,38], microchannel chamber by Lu et al. [39]) and/or are limited in space and time of observation (OMEGA^4^ co-culture device, Wittig et al. [27]). The lack of a suitable in vitro test system is to be remedied by the NOCs in combination with mini-osmotic pumps suitable for in vivo applications. In the NOCs, the effect of drugs and drug delivery strategies on neuronal survival and (guided) neurite outgrowth can be investigated under conditions that imitate the in vivo situation more accurately than conventional cultures. The NOC is built of two compartments mimicking the cochlea, albeit a simpler version, with a spatial distance between drug source and target neurons. The following requirements necessary for investigations of chronical pharmacotherapies on SGNs are addressed by the NOC design: the integration of a long-term drug delivery source, culturing non-dissociated SGEs for neurite regeneration, an open top design, taking the anatomy of the human cochlea into account, a quick and cheap production allowing a high adaptability to novel designs, and an easy handling of the NOCs during cultivation. To support good handling of the SGEs and the formation of a diffusion gradient, a fivefold increase of the reported distance between the center of the RC and the CI electrode array [34] are chosen for the NOC dimensions. For pharmacokinetic and -dynamic studies, a culture chamber that mimics the exact human anatomical situation in terms of dimensions, distances, and physiological barriers would be optimal. However, since the inner ear, and especially the ST and the RC, are very small structures, the anatomical dimensions do not allow handling of cultivated cells in such a small working space. A sufficient medium supply and an observation of the cells during culturing would also hardly be possible. Therefore, we chose a compromise between anatomical correctness and manageability of the cell culture device. The NOC’s open top design enables a placement of the SGEs with forceps from above and eases access to cells and for medium change. In combination with the glass bottom, a constant microscopical observation of the cells during the experiment is possible. Drug delivery via catheter and mini-osmotic pump to the NOC, in order to mimic an intracochlear drug delivery route, is enabled by the added inlet. The positioning of the inlet in the lateral wall of the ST-compartment supports a formation of a diffusion gradient, since a distance of about 10 mm to the SGEs in the RC-compartment has to be overcome. In addition, combining other research approaches, such as electrical stimulation, with a CI is simplified by this inlet position. One great advantage of the design is that the NOC enables the examination of guided neurite outgrowth by investigating the orientation of the outgrown neurites towards or away from the drug source. Another advantage is the customizability of the NOC because of the 3D printing technology that is used. The CAD data can easily be modified for adjustments of the NOC for different experimental purposes. The fused deposition modeling technology convinces with a high-quality production that is also very quick and cheap (~33 min and ~6 cent per NOC). The printed material PLA is a polyester of lactic acid and known for its biocompatibility [40,41], as is the silicone [42,43,44] used for the final coating. Previous studies proved that the used PLA does not affect the viability of neuronal cells and is therefore used for a variety of in vitro studies [45,46,47]. However, the contact of the medium with PLA is very limited and takes place only in the inlet, which is not covered by silicone. The typical outgrowth of non-neuronal cells in a radius around the SGEs and the observed neurite extension in all treatment groups (Figure 6) can be rated as indicators for a good compatibility of the NOC materials. Compared with the previously used UV curing silicone for NOC printing, the PLA enables a clean print of the inlet and provides a stable form. The final coating with a thin layer of hydrophobic silicone reliably prevents absorption of the medium or NTFs in the slightly porous PLA structure.

The size of the NOC allows the culturing of the entire SG. Due to its spiral shape, a correct, de-coiled placement and attachment in the NOC is difficult. Embedding the SG in a matrix-like collagen could support a culture of the whole ganglion but could also influence the factor distribution and lead to three-dimensional cell and neurite growth, making microscopic observation and neurite analysis difficult. Therefore, we decided not to use it in the present setup, but the option of a 3D culture should be addressed in future studies. Tissue adhesion on the glass coverslips is improved by cutting the SG into five pieces, by coating the glass with ornithine and laminin, by leaving the NOCs at room temperature for 30 min without any movements after SGE positioning, and by pre-cultivating the SGEs. Not all SGEs adhere where they are originally placed, regardless of the experimental group. Detachment of explants is a known obstacle when working with non-dissociated SG tissue [48,49,50]. Nevertheless, the entire SG is initially seeded in the NOC and SGEs are subsequently analyzed without distinguishing between basal, middle, and apical turn. Therefore, they naturally vary in size between the wide basal and the small apical region, which results in a wide dispersion of the SGE areas that have a clear similarity in all groups (Figure 7A). Thus, all SGEs have the same prerequisites for comparability of neurite outgrowth. In order to exclude a dependence between the number of outgrown neurites and size of SGEs, the number of neurites is calculated per 100,000 µm^2^ explant area.

The setup (Figure 3) with the mini-osmotic pumps, the modified six well plates, and the pedestals for the NOC elevation is very satisfactory in terms of handling and functionality. It allows the performance of four experiments at the same time (e.g., test of two factors and two controls) and a factor release over several weeks. Depending on the used pump, a drug release period of up to 6 weeks (Alzet model 2006) could be observed in the presented setup. The used mini-osmotic pumps consist of an inner drug reservoir, an osmotic layer, and an outer semipermeable membrane. The mode of action is based on an osmotic pressure difference between the osmotic layer and the environment in which the pump is placed. Water flows into the pump through the membrane, expanding the osmotic layer and compressing the drug reservoir. This leads to displacement, and therefore, release of the drug, whereby the permeability of the membrane determines the delivery rate [51]. The biological efficacy of a compound decreases over time which can be due to, for example, degradation in the higher “body” temperature or clumping in the pump. Therefore, it is important to ensure a consistent effect or detect a declining efficacy before performing in vivo experiments. The presented NOC setup is designed to investigate the question of remaining factor activity over a long (up to 6 weeks) time period. By replacing the NOCs after some days with ones that have fresh SGEs, tested factors can be observed over a long period to determine the timing of a decrease in effectiveness. Thus, the NOCs are a great tool for this purpose and can inter alia improve animal testing according to the 3Rs (Replace, Reduce, Refine).

The NOC setup with the pumps is validated using NT-3 [26,27,28,29,30] and CNTF [23,24,25,52] as known neuroprotective and neuritogenic factors. A treatment of the SGEs in the NOCs with NT-3 results in a significant increase in the number of regenerated neurites (Figure 7B), neurite length (Figure 8A), and thus neurite coverage area (Figure 9A), compared to the other groups. This proves the functionality of the setup. However, no change in the directional growth of the neurites indicating a guidance to the NT-3-delivering pump is detected (Figure 7C). Even though neurite outgrowth in direction to the pump is higher for NT-3-treated SGEs compared with NC and CNTF, there is no difference within the NT-3 group itself (Figure 8B). This result is in contrast to the neurite attraction by NT-3 infusion published by Wittig et al. [27]. A possible explanation for this discrepancy is the low number of three analyzed SGEs with regenerating neurites in the free-choice area and only six rated neurites in the whole study. In addition, the used part of the SG is not described in detail, whereby the action of the neurotrophins brain-derived neurotrophic factor and NT-3 on the SGNs is known to be location-dependent in the SG [53,54]. Since the whole SG is cultured and analyzed in the NOCs, a possible region-dependent responsiveness to NT-3 could have been masked. Although the NT-3 amount delivered to the SGEs in the NOCs is based on the one described by Wittig et al. the delivery rate of our pumps is much lower (0.5 µL/h compared with 15–30 µL/h) and the distance to the drug source is much larger, which possibly reduces the guidance efficacy. Compared with NT-3, CNTF fails to increase neurite outgrowth of SGEs in the NOCs. A concentration of 100 ng/mL CNTF on dissociated SGNs is able to promote neurite outgrowth significantly [23]. Therefore, this concentration is used as a basis for the calculation of the pump release. In purely mathematical terms, this concentration is reached in the NOC within 30 h by pump release, theoretically giving the opportunity to form a chemical gradient. One reason for the lack of an increase in neurite outgrowth may be that the used exogenous CNTF protein has a different effect on dissociated SGNs than on non-dissociated SGEs [35]. Hartnick et al. showed that there was a neuritogenic effect of CNTF for both dissociated SGNs and micro explants [25]. However, the age of the used animals differed (5 days compared with 2–4 days), which can influence neurite regeneration by factor treatment [55,56]. They also refreshed the medium with NTFs every other day. Another reason for the deficiency of neurite outgrowth increase could be that the chosen CNTF concentration and release rate are too low. Usually, NTFs are applied as a single-dose treatment (burst release) and are refreshed by medium change if needed, due to time of cultivation. The NOC setup is chosen to mimic the in vivo situation with a continuous release of NTFs and no refreshing. In this context, our results could indicate that CNTF loses its bioefficacy very quickly when chronically released by pumps, although no significant differences between the first and second pump-cycle are evident. On the other hand, the continuous release, resulting in an accumulation over time, could possibly even be the reason for the shorter regenerated neurites compared with the NC group. A guided neurite outgrowth towards the CNTF-releasing osmotic pump cannot be determined either (Figure 7C).

A daily medium change (MC) has no effect on the SGEs of the NC and the NP condition, but it neutralizes the neuritogenic effect of the NT-3 delivery (Figure 10B). This indicates that the continuous release and accumulation of NT-3 is necessary for the detected increased neurite outgrowth. The noticeable, significantly shorter neurite length in those directed toward the pump (NT-3 MC +), compared with those facing away from the pump (NT-3 MC −), may be based on mechanical stress due to turbulences appearing when new medium is added to the inlet side of the ST-compartment (Figure 11B). However, this effect is restricted to the in vitro situation, since the neurites of the SGNs in the cochlea are protected from quick liquid diffusion by their location in the osseous spiral lamina (Figure 2A). Interestingly, in the NOC conditions supplying AP via the pumps (NC), significantly increased neurite lengths are detected when compared to the NP group (Figure 8A). This could be due to the calcium contained in the AP which may elicit neurite outgrowth [57,58].

In this study, we focused on the integration of an established and common in vivo test system for long-term drug-release [8,12,13,59,60], the mini-osmotic pumps from Alzet, and the observation of the biological effect of NTFs on neurite growth. An analysis of the NTF distribution and concentration gradients in the NOC (e.g., by fluid dynamic modeling) is not performed. Especially in terms of guided neurite growth, this is of great interest for future experiments. For example, the NTF concentration at the inlet, where the NTF is released, could be compared with those that are close to the SGEs.

## 5. Conclusions

A new culture device is designed and a simple setup is established allowing long-term drug delivery by mini-osmotic pumps to inner ear cells. As part of this study, the setup is validated with two test factors known to support neurite outgrowth. The results show that the NOCs are very suitable for culturing SGEs in combination with pump-based drug delivery, and they allow easy handling of the cells for analysis. Pump-released NT-3 proved the concept of detecting NTF-induced neurite outgrowth in the NOCs, except for neurite guidance. The opportunity to observe the biological efficacy of factors delivered by osmotic pumps over weeks to regularly replaced inner ear tissue is an outstanding advantage of the presented NOC, mimicking the in vivo situation of long-term drug delivery. Due to the remarkable adaptability of the chamber, adjustments to new research questions are easy to implement. Further growth factors could be tested, and matrices or barriers could be added in the canal region. In addition, a combination with CI electrodes for combined electrical stimulation with drug release, as well as an adaption to other forms of drug delivery is possible. This in vitro model is particularly suitable for investigations on how to bridge the anatomical gap between the SGNs and the CI, from which a significant improvement of the CI function is anticipated.

## Figures and Tables

**Table 1:** Technical Data of one Printing Process producing 11 NOCs.

Printing Technology	Fused Deposition Modeling
Printing time	5 h 25 min printing process, 6 h including pre- and post-set up such as removing residuals of material and checking the consistency of the inlet, ~33 min/NOC
Nozzle temperature	210 °C
Nozzle diameter	0.4 mm
Layer thickness	0.06 mm
Fill density	100%
Overlap	25%
Material amount	Orange-colored PLA, ~18 g (excluding PLA extrusion at the start of the process), ~1.64 g/NOC
Material costs	~25.95 €/750 g filament role (excluding shipment), ~6 cent/NOC

**Table 2:** Number of Experiments per Treatment Condition.

Treatment Condition							
	NC	NP	CNTF	NT-3	NC MC	NP MC	NT-3 MC
**N**	11	8	7	4	5	5	5

NC = negative control; NP = no pump; CNTF = ciliary neurotrophic factor; NT-3 = neurotrophin-3; NC MC = negative control with medium change; NP MC = no pump with medium change; NT-3 MC = neurotrophin-3 with medium change.

**Table 3:** Neurite Length (µm) independent of the Direction of Outgrowth (without MC).

	NC	NP	CNTF	NT-3
**Minimum**	35	20	53	25
**Maximum**	2602	3631	2377	2403
**Mean**	717 ± 434	670 ± 489	603 ± 336	799 ± 475

**Table 4:** Neurite Length (µm) dependent on the Direction of Outgrowth (without MC).

	NC +	NC −	CNTF +	CNTF −	NT-3 +	NT-3 −
**Minimum**	35	47	59	53	25	37
**Maximum**	2077	2602	1609	2377	2344	2403
**Mean**	701 ± 433	732 ± 436	597 ± 311	614 ± 363	814 ± 474	784 ± 476

## Data Availability

All data are available upon request.
